# Gaze following in an asocial reptile (*Eublepharis macularius*)

**DOI:** 10.1007/s10071-018-1230-y

**Published:** 2018-12-22

**Authors:** Joe Simpson, Sean J. O’Hara

**Affiliations:** School of Environment and Life Sciences, University of Salford, Peel Building, Salford, M5 4WT UK

**Keywords:** Gaze following, Reptile cognition, Leopard gecko, Gaze sensitivity

## Abstract

Gaze following is the ability to utilise information from another’s gaze. It is most often seen in a social context or as a reflexive response to interesting external stimuli. Social species can potentially reveal utilisable knowledge about another’s future intentions by attending to the target of their gaze. However, in even more fundamental situations, being sensitive to another’s gaze can also be useful such as when it can facilitate greater foraging efficiency or lead to earlier predator detection. While gaze sensitivity has been shown to be prevalent in a number of social species, little is currently known about the potential for gaze following in asocial species. The current study investigated whether an asocial reptile, the leopard gecko (*Eublepharis macularius*), could reliably use the visual indicators of attention to follow the gaze of a conspecific around a barrier. We operated three trial conditions and found subjects (*N* = 6) responded significantly more to the conspecific demonstrator looking up at a laser stimulus projected onto an occluder during the experimental condition compared to either of two control conditions. The study’s findings point toward growing evidence for gaze-following ability in reptiles, who are typically categorised as asocial. Furthermore, our findings support developing comparative social cognition research showing the origins of gaze following and other cognitive behaviours that may be more widely distributed across taxonomic groups than hitherto thought.

## Introduction

Gaze following is the ability to coordinate one’s gaze with that of another individual (Butterworth and Jarett 1991). Growing evidence points toward social factors often being responsible for influencing gaze-following responses (Frith and Frith 2008; Goossens et al. 2008; Jones et al. 2010; Kano and Call 2014). Thus, it may not be always modulated by a reflexive biological response alone, demonstrated in relation to seeing another individual viewing an interesting stimulus (cf. Senju et al. 2004). At its most cognitive, gaze following can be considered to be one key component of possessing a theory of mind (Baron-Cohen and Cross 1992; Penn and Povinelli 2007; Call and Tomasello 2008), i.e., when viewed in the context of recognising that others have an altered knowledge state that differs from one’s own, when they can see things that you cannot. In its simpler form, the propensity to gaze follow has been described as a survival adaption that allows for more efficient food locating or predator detection (Tomasello et al. 1998; Itakura et al. 1999; Bugnyar et al. 2004; Amici et al. 2009). Since the early 1970s, there has been evidence accumulated for effective use of gaze following in a plethora of bird species able to evaluate the risk from potential predators in terms of proximity (Ydenberg and Dill 1986; Palleroni et al. 2005) or whether a predator is about to attack (Gallup et al. 1972; Carter et al. 2008). In terms of survivability, having the capability to read subtle cues of predators through gaze following would be beneficial. Carter and colleagues (2008), for instance, found that European starlings (*Sturnus vulgaris*) could effectively utilise human predator gaze cues to coordinate their behaviour, i.e., reduce or increase the frequency of feeding based on whether the predator’s gaze was toward the starlings or averted.

A swathe of primate-focussed studies have reported positive findings in the great apes (Call et al. 1998; Povinelli et al. 1999; Schmid et al. 2017), Old World monkeys (Vick and Anderson 2003; Shepherd et al. 2006; Goossens et al. 2008; Teufel et al. 2010; Micheletta and Waller 2012; Hopper et al. 2013; Overduin-de Vries et al. 2014), New World monkeys (Burkart and Heschl 2006) and lemurs (Anderson and Mitchell 1999). Notwithstanding, there is growing evidence of gaze following beyond primates, with recent confirmation reported in birds such as corvids (Bugnyar et al. 2004; Schloegl et al. 2007; Schmidt et al. 2011; Tornick et al. 2011), the Northern bald ibis (Loretto et al. 2010) and some species of passerines (Watve et al. 2002; Jaime et al. 2009). In non-primate mammals, there has also been positive findings, suggesting other mammalian species can coordinate their gaze orientation toward a given location (domestic goats: Kaminski et al. 2005; dolphins: Pack and Herman 2006; canids [domestic dogs and wolves]: Miklósi et al. 1998; Range and Viranyi 2011), and limited anecdotal evidence of at least some gaze sensitivity in spotted hyenas *Crocuta crocuta* (Holekamp et al. 2007). Those findings are telling us gaze-following ability falls wider from its putative ‘origin’ than was previously supposed. They also support the notion that the cognitive mechanisms that underpin gaze following have evolved via convergent evolution. Indeed, Seed et al. (2009) and others postulated that the evidence for comparable intelligence in humans, non-human primates and corvids suggests intelligence evolved independently across taxonomic groups in taxa facing similar cognitive challenges in similar external environments, i.e., sociality, predation, and mate finding (Emery and Clayton 2004; Seed et al. 2009). Challenges, however, have only to be similar not the same. Thus, asocial species could find themselves confronting similar cognitive challenges at those times when needing to find a mate, for example. The diversity of taxa now known to gaze follow tells us taxonomic distance ought not be a barrier to research in the study of gaze following, and to develop our understanding of the potential origins of cognitive abilities that facilitate gaze following, it would be appropriate to compare gaze following across different taxonomic groups (Wilkinson et al. 2010; MacLean et al. 2012).

One taxonomic group that has been largely overlooked in gaze-following research is reptiles (Burghardt et al. 1977; Kis et al. 2015; Wilkinson 2016). This is perhaps partly due to their categorisation as asocial animals (Doody et al. 2013), since gaze following is considered advantageous in those taxa where conspecifics routinely interact. Recently, however, the existing taxonomically constrained hypothesis has undergone challenge with gaze following reported in red-footed tortoises (Wilkinson et al. 2010) and bearded dragons (Siviter et al. 2017). Such evidence has led to speculation that gaze-following ability may have deep evolutionary origins, originating in the common ancestor shared with birds and mammals 280 MYA (Wilkinson et al. 2010; Alföldi et al. 2011; Doody et al. 2013). If this were to be true, it would predict the existence of a more widely distributed potential for gaze following, prevalent across taxonomic groups, including reptiles (Wilkinson and Huber 2012). Different reptile species need to be studied to determine the true extent of such traits within this taxonomic group (Matsubara et al. 2017) to unpick whether they are limited to few species or more widely distributed across reptiles. Sourcing the evolutionary origins of any trait is perhaps best achieved by adopting a comparative approach. Giving greater attention to different taxonomic groups such as reptiles has the potential to enhance our understanding of the origins of gaze following and other cognitive traits. It is important to document differences between closely related and distant species and by showing differences between context, habitat or other social and environmental factors, we can better understand the evolution and function of gaze following.

Here, we test for the gaze-following potential of leopard geckos (*Eublepharis macularius*). The leopard gecko is a crepuscular species of the family Eublepharidae, which comprises 30 different species (Gamble et al. 2011). They are typically asocial, rarely socially interacting with conspecifics beyond courtship or food or mate competition (Gamble et al. 2011; Srinivasulu and Srinivasulu 2013). Leopard geckos inhabit rocky, arid deserts and grasslands across Afghanistan and north-west Pakistan as well as parts of Iran (Srinivasulu and Srinivasulu 2013). Although little has been studied on the visual perception in leopard geckos, investigation has revealed the species relies heavily on socio-chemical communication to differentiate between the sexes. Females emit specialised pheromones released from pores on their skin that signal to males that they are female. In the absence of female pheromones, males act aggressively towards unfamiliar individuals irrespective of gender (Mason and Gutzke 1990). Although leopard geckos are solitary, they can be housed together in captivity. Squamates show visual sensitivity in relation to predation risk, actively averting their gaze when approached and stared at by a human ‘predator’ coming directly toward them but not exhibiting this behaviour when approached diagonally (Burger et al. 1992; Elmasri et al. 2012; Sreekar and Quader 2013). Siviter et al. (2017) recently showed that bearded dragons (*Pongo vitticeps*) are able to follow the gaze of another individual around a barrier into distant space. Therefore, with evidence for some level of gaze-associated cognitive ability in other lizard species, leopard geckos provide a useful comparison to help reveal the extent of social cognition in otherwise asocial reptiles. Furthermore, unlike other nocturnal or crepuscular species, leopard geckos have nocturnal colour vision. They possess multiple cones within the retina allowing them to see colour in darkness (Roth and Kelber 2004). Accordingly, leopard geckos are able to distinguish different colour shades and see ultraviolet light, but not red light, in total darkness. This apparent visual sensitivity to different lights make the leopard gecko an ideal species with which to experimentally test their gaze-following potential.

## Materials and methods

### Study subjects

Seven captive-bred leopard geckos were used in the study. The subjects comprised five females and two males, whose ages ranged from 2 (juvenile) to 14 years (Table 2). Subjects here were kept in five heated vivaria (constant temperature of 28 ± 2 °C), housed in duos (LB and SK; DO and JY) or singly (LO, Un-named 1, Un-named 2) at the study location, Broomfield College Animal Care Centre, Derby College, U.K. DO and JY had been kept together for 2 years prior to the onset of the study. LB and SK were paired temporarily for the study period. The three singly housed individuals had also been kept singly prior to the study period. Subjects had no previous exposure to individuals other than where stated above.

Subjects were fed twice daily at 07.00 and 13.00. Morning trials were conducted post 9 am and afternoon ones post 2.30 p.m. Since leopard geckos are crepuscular, we conducted our trials with the lights turned off in the experiment room, relying only on lighting from those vivaria present. All of our subjects were experimentally naïve.

### Apparatus

**Fig. 1:**
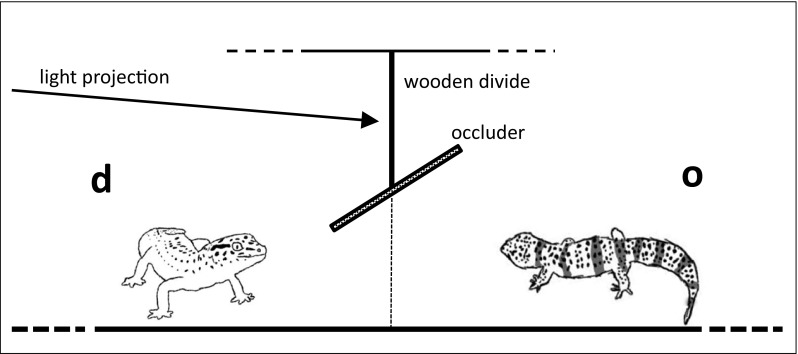
The experimental set up. Tanks were set end to end allowing ground-level visual access to the demonstrator (d) by the observer (o). Access to where the demonstrator looked (site of laser projection) was blocked, for the observer, by the occluder. During the trials, the lids of the tanks were fastened

Experimental arenas comprised two glass-sided fish tanks (35 × 25 × 24 cm), positioned horizontally and placed end to end (Fig. 1). The adjoining ends allowed clear visibility into the other tank from ground level to 6 cm high, sufficient to easily view a conspecific through. A wooden occluder (25.5 × 19.7 cm) was set in an angled positioned between the two tanks. The lids of the tanks were made of black plastic comprising a transparent, rectangular, plastic flap that could be lifted to reach in to access the subjects. The lid and the flap were kept closed during the trials. To reduce the possibility of laser light reflection onto the flap, the underside of the flap was covered with newspaper. The three sides of the demonstrator’s tank were also covered with newspaper to reduce any reflected laser light against the sides of the tank. A StreamLight Stylus Pro^®^ Penlight (model no. 66124) with a green laser light was shone onto the occluder from outside the tank, behind on the demonstrator’s side. Therefore, the demonstrator, but not the observer, was able to see and orientate toward the laser light. The laser was handheld by the experimenter, so that the laser could be projected freely across horizontal occluder rather than just being shone in a fixed position. Switching the laser pen on/off appeared to not generate any audible interference.

### Pre-testing

Pre-testing trials were undertaken to select a demonstrator for the main study. Each subject was tested in five trials over a 2-day period (July 2017). Because of the potential for male aggression toward unfamiliar individuals (Mason and Gutzke 1990), we tested only our five females for the role of demonstrator. We used the criterion of most “look ups” to select the testing phase demonstrator. Adult female (LB) was chosen, since she reliably looked up (head and neck orientation) toward the laser stimulus. The remaining six subjects were therefore assigned the role of observer.

### Looking up behaviour

We recorded the looking up behaviour performed by the demonstrator and the following response by the observer to the demonstrator looking up at the laser. When defining looking up behaviour in leopard geckos, our pre-testing observations established the presence of three different forms of “looking up” behaviour (Table 1).

**Table 1:** Definitions of “looking up” behaviour in leopard geckos

Look up behaviour	Description
Look up stationary	Head and neck extended upward orientation; no movement toward front of tank
Look up and move forward	Head and neck extended upward orientation; movement toward front of tank
Look up and climb forward	Head and neck extended upward orientation; movement toward front of tank and an attempt to climb front of tank

### Procedure

The main testing phase of the study was conducted over a 3-week period (July–Aug. 2017) and we limited trials to Mondays, Wednesdays and Fridays to reduce the potential for habituation that daily testing might have induced. Subjects were each exposed to three testing periods: the experimental condition and two controls. Each trial condition lasted 60 s. We randomised presentation order to reduce the possibility of order effects. All subjects received the same number of trials with each of the six given three trials of the corresponding conditions (i.e. 3 × 3 trials each).

### Experimental condition

In the experimental condition, a gaze-following response was recorded if the observer extended their head and neck toward the stimulus or climbed the tank and then extended their neck and head toward the stimulus (see e.g., Loretto et al. 2010; Wilkinson et al. 2010; Table 1). A “look up” response was scored for any look up with a minimum duration of 10 s. Since the laser was being shone onto the top section of the tank above the angled occluder, to attempt to orientate toward where the demonstrator was looking, the observer’s looking up action was unambiguous.

### No laser control

In this trial, to rule out the possibility the observer was simply looking up when faced by a conspecific, no laser was projected and the demonstrator, although present, was not encouraged to look up (no light beam). We terminated (and re-ran) the trial if the demonstrator did not refrain from looking upward for the full 60 s. For any of the trials that were prematurely terminated, these trials were not included.

### No demonstrator control

In the no demonstrator control condition, the demonstrator was removed to test for the possibility that the observer was able to see the laser pointer and the light stimulus, rather than it being the demonstrator’s actions, that was cueing their looking response. The condition was identical to the experimental condition except with no demonstrator present. The observer was kept still in their tank facing the adjoining tank, while a laser was shone onto the occluder. A look up response could imply the stimulus was visible to them. A null response was recorded if the observer failed to look up.

### Statistical analysis and inter-rater reliability

We used a repeated-measures study design based on the combined number of “look ups” exhibited by each individual across all three trials. Due to a limiting sample size, we applied a Friedman’s non-parametric test to detect the difference in look up behaviour under different test conditions across each of the individuals. We employed Bonferroni-corrected Wilcoxon signed-rank tests for post hoc testing. The effect size was calculated using *r* = z/√*N*. According to Cohen (1988), the effect size threshold of *r* = 0.1 is small; *r* = 0.3 is medium and *r* = 0.5 is large. The statistical tests were conducted using IBM SPSS (version 24.1).

To validate our coding procedure, we ran a separate series of trials over 1 day in August 2017. Those trials were for used for inter-observer reliability purposes only and are not included in the data set presented. Trials followed the same procedure of the experimental condition and the two control conditions. Testing was filmed using an Apple iPad 2 fixed to a stand at the back of the observer’s tank, facing the adjoining tank. Videos were labelled according to trial type. An independent coder (blind to experimental condition) was trained to recognise ‘following’ responses using still images showing the different sorts of responses used by leopard geckos when gaze following. These included head and neck orientation toward the occluder and attempting to climb the front of the testing tank coupled with head and neck alignment toward the light stimulus (Table 1). The primary coder (JAS) coded 100% of the 31 videos. The independent coder then coded 16 randomly selected videos. Inter-rater reliability testing was performed to determine the level of uniformity between the two coders. Inter-rater reliability was high with 80% agreement in the responses recorded. Applying Cohen’s *k* to determine the level to which this agreement could be attributed to chance highlighted a moderate to good level of agreement between the two coders *k* = 0.57, *p* < 0.0001.

## Results

**Fig. 2:**
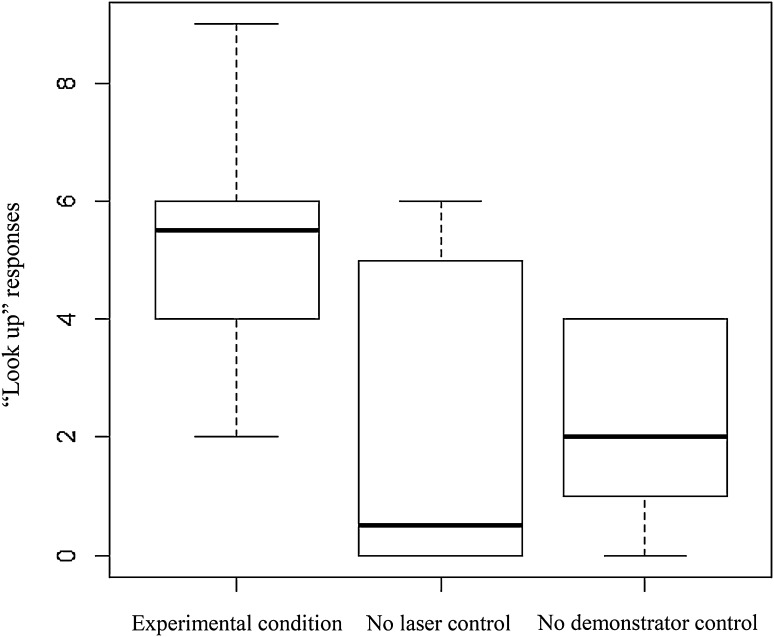
The number of “looking up” responses by testing condition. Medians and interquartile ranges are indicated

The combined number of look ups observed in each subject across the different test conditions is presented in Table 2. The median combined number of look up responses across all subjects per test condition is shown in Fig. 2. All six subjects demonstrated a gaze-following response in the experimental condition and only two of the six responded under all test conditions. Four of the six subjects scored more responses in the experimental condition compared to the two control conditions.

**Table 2:** Name, sex, age and size, of the leopard geckos (*Eublepharis macularius*) used in the current study together with performance of subjects under the three trial conditions

Gecko ID	Sex	Age (years)	Size (cm)	Housed with	Experimental condition	Control 1: no laser	Control 2: no demonstrator
LB*	F	12	19	SK	–	–	–
LO	M	14	22.9	–	2	0	2
DO	F	2	17.5	JY	6	5	4
SK	M	10–12	18.5	LB	5	1	4
Un-named 1	F	2	19	–	6	6	0
JY	F	2	18	DO	9	0	1
Un-named 2	F	2	17.6	–	4	0	2

Numbers indicate the number of times an individual responded to the given condition

*Demonstrator

The Friedman’s test of these data identified a significant difference in look up behaviour under the three test conditions (*X*^*2*^ = 7.182, *df* = 2, *p* = 0.028). Median (IQR) “look up” behaviour for the experimental condition, the no laser control and the no demonstrator control were 5.5 (5.50–6.75), 0.5 (0–5.25) and 2.0 (0.75–4.0), respectively. Post hoc analysis using Bonferroni-corrected Wilcoxon signed-rank tests revealed that subjects responded significantly more in the experimental condition than in both the no laser control (experimental—no laser: *Z* = − 2.032, *p* = 0.042, *r*= − 0.59) and the no demonstrator control conditions (experimental—no demonstrator: *Z* = − 2.032, *p* = 0.042; *r* = − 0.59), while there was no significant difference between responses to the no laser and no demonstrator controls (*Z* = − 0.632, *p* = 0.527; *r* = − 0.18).

## Discussion

The current study’s findings have revealed the first recorded occurrence of gaze-following behaviour in leopard geckos and, to our knowledge, only the third time evidence for gaze-following abilities have been found in reptiles in general (Wilkinson et al. 2010). Subjects in the current study reliably followed the gaze of a conspecific around a barrier. The findings lend further support to the claim that gaze-following behaviour is not limited to humans, non-human primates and some birds (Barth et al. 2005; Bugnyar et al. 2004; Tornick et al. 2011) but extends to other taxa including some groups of reptiles.

Wilkinson et al. (2010) suggested younger tortoises in their study were less responsive to a laser stimulus than adults. The inference being there may be interesting implications for the ontogeny of gaze-following ability in reptiles. Although our data cannot be used for statistically significant evaluation, there were some observable differences in the performance of the leopard geckos that may support a learning proposition, with younger geckos responding more in the experimental condition than adults. The sample size was, however, sufficient to demonstrate gaze following in leopard geckos and their postural response behaviour could be clearly differentiated from the kind of tail fanning or left to right head shifting characteristic of leopard gecko mating behaviour. Our subjects were sensitive to, and behaviourally responded to, the gaze orientation of an unfamiliar conspecific. However, while it has been possible to control for postural orientation in other taxa (e.g., chimpanzees: Hare et al. 2006) to ensure it is the gaze rather than the posture that is being followed, this has yet to be done in reptiles. Since looking behaviour in leopard geckos is also accompanied by postural orientation, it may be that movement detection, rather than gaze sensitivity, led to observers’ looking behaviour. We are unable to rule out this possibility here and future studies should employ designs that can better tease this apart. Also, seldom do behavioural studies of animal cognition measure brain activity so as to infer internal mental states. Thus, while our behavioural observations of subjects, and the nature of the rather precise directional orientations they took to look towards where the demonstrator was gazing, both suggest these were genuine gaze-following recordings, it is also plausible that some recorded look ups could have been motivated by other factors. However, given that more look ups were observed in the experimental condition than in either of the controls makes that explanation unlikely.

Since gaze following is expected to be beneficial in socially interacting species, the ability has been linked to social exposure. In Wilkinson et al’s (2010) red-footed tortoise study, they found tortoises reliably followed the gaze of conspecifics around an occluder, and did so following an extended period of social exposure (the subjects were housed all together for 6 months). The social exposure hypothesis thus provides one explanation as to why those tortoises may have performed above expectation. The current study, however, is unable to highlight a social exposure/positive performance link in a gaze-following task. Here, one would have expected the individual (SK) socially housed with the demonstrator (LB) to out-perform other subjects, but he is ranked as only the median responder. The performance of the other socially housed pair (JY/DO) was not any more impressive than singly housed subjects. Thus, while a priori exposure to the demonstrator may have facilitated gaze-orientated responses in previous studies (e.g., Burger et al. 1992; Wilkinson et al. 2010), our results are unable to lend support to the proposition that past exposure enhances learning performance. Specific tests with larger sample sizes will be needed to discern this.

In seeking the evolutionary origins of gaze following, Wilkinson et al. (2010) have argued that gaze-following abilities may be rooted in the phylogenetic split at the amniotic level, around 280 MYA, which represents the divergence between reptiles, birds and mammals from a common ancestor. So far, reptilian research has uncovered gaze sensitivity and gaze following in squamates (Burger et al. 1992) and chelonians (Wilkinson et al. 2010). Thus, one possibility is that gaze following originated in a common ancestor of birds and mammals that split from reptiles around the time of the split between the Squamata and Chelonii, explaining why lizards and tortoises have demonstrated similar abilities. However, more work on species within Reptilia is required to confirm the plausibility of this hypothesis. Currently, an alternative to the deep origin hypothesis is that gaze-following ability has arisen through convergent evolution. Future studies that add to the diversity of gaze-following taxa can further add to our understanding of how similar social contexts or habitat attributes may have influenced or seeded the evolution of gaze-following abilities.

The current study reveals findings that further extend our knowledge of reptile cognition, which has lagged behind other taxonomic groups. It remains, however, in its early stages. A better understanding of how behaviour is distributed across the natural world can only be achieved through taxonomic breadth and reptile cognition research offers great potential. Since its initial discovery in Reptilia, we are now learning gaze-following ability is not unique to Chelonii. How far it might extend remains an open question.
